# The diversity of the N_2_O reducers matters for the N_2_O:N_2_ denitrification end-product ratio across an annual and a perennial cropping system

**DOI:** 10.3389/fmicb.2015.00971

**Published:** 2015-09-24

**Authors:** Luiz A. Domeignoz-Horta, Aymé Spor, David Bru, Marie-Christine Breuil, Florian Bizouard, Joël Léonard, Laurent Philippot

**Affiliations:** ^1^INRA, UMR 1347 AgroécologieDijon, France; ^2^INRA, UR 1158 AgroImpactLaon, France

**Keywords:** *nosZ*, greenhouse gas, agroecology, diversity, nitrous oxide, agricultural practices, nitrogen cycling

## Abstract

Agriculture is the main source of terrestrial emissions of N_2_O, a potent greenhouse gas and the main cause of ozone layer depletion. The reduction of N_2_O into N_2_ by microorganisms carrying the nitrous oxide reductase gene (*nosZ*) is the only biological process known to eliminate this greenhouse gas. Recent studies showed that a previously unknown clade of N_2_O-reducers was related to the capacity of the soil to act as an N_2_O sink, opening the way for new strategies to mitigate emissions. Here, we investigated whether the agricultural practices could differently influence the two N_2_O reducer clades with consequences for denitrification end-products. The abundance of N_2_O-reducers and producers was quantified by real-time PCR, and the diversity of both *nosZ* clades was determined by 454 pyrosequencing. Potential N_2_O production and potential denitrification activity were used to calculate the denitrification gaseous end-product ratio. Overall, the results showed limited differences between management practices but there were significant differences between cropping systems in both the abundance and structure of the *nosZII* community, as well as in the [rN_2_O/r(N_2_O+N_2_)] ratio. More limited differences were observed in the *nosZI* community, suggesting that the newly identified *nosZII* clade is more sensitive than *nosZI* to environmental changes. Potential denitrification activity and potential N_2_O production were explained mainly by the soil properties while the diversity of the *nosZII* clade on its own explained 26% of the denitrification end-product ratio, which highlights the importance of understanding the ecology of this newly identified clade of N_2_O reducers for mitigation strategies.

## Introduction

Nitrous oxide (N_2_O) is one of the six gases subject to restriction by the Kyoto Protocol, which aims at reducing anthropogenic greenhouse gas (GHG) emissions. N_2_O is both directly and indirectly of importance for the Earth's climate. Firstly, it is a potent greenhouse gas with a long life time of 110 years and a global warming potential 298 times that of carbon dioxide on a 100-year time scale and per unit of weight. Thus, N_2_O is the third most important GHG contributing to about 10% of annual global warming (Bates et al., 2008; Thomson et al., 2012). Secondly, after the success of the Montreal Protocol for phasing out chlorofluorocarbons (CFCs), N_2_O is today the dominant ozone-depleting substance (Ravishankara et al., 2009). The atmospheric concentration of N_2_O has been rising over the past 100 years resulting in a concentration 19% higher than pre-industrial levels (Montzka et al., 2011) with an estimated increase of N_2_O emissions of up to 60% by 2050 (relative to 1900 values) (Bouwman et al., 2013).

N_2_O emissions are, to a great extent, the result of microbial processes such as nitrification and denitrification. However, denitrification is also the only known sink for N_2_O. Denitrification is a microbial respiratory pathway through which soluble forms of nitrogen, i.e., nitrate (NO${}_{3}^{-}$) and nitrite (NO${}_{2}^{-}$), are sequentially transformed into NO, N_2_O and N_2_ gases via four enzymatic systems (Tiedje et al., 1982; Zumft, 1997). The reduction of soluble NO${}_{2}^{-}$ into NO and N_2_O is catalyzed by copper- or *cd*_1_- nitrite reductases and nitric oxide reductases, respectively (Zumft, 2005). Nitrous oxide reductase, whose catalytic subunit is encoded by the *nosZ* gene, is the last enzyme of the pathway. It converts the GHG N_2_O into inert N_2_, which accounts for 78% of the atmospheric gases, and is, therefore, the key enzyme involved in the N_2_O sink. It is now recognized that denitrification is a modular process (Graf et al., 2014). Thus, while some microorganisms harbor all denitrification enzymes and can potentially perform the complete pathway, others either lack the nitrous oxide reductase gene and produce only N_2_O as the denitrification end product (Philippot et al., 2011), or are only able to reduce N_2_O (Sanford et al., 2002).

Recent studies have identified a previously undescribed *nosZ* clade, herein after named *nosZII*, which is diverse and widespread in the environment (Sanford et al., 2012; Jones et al., 2013; Orellana et al., 2014). Genome analyses showed that an important fraction of the microorganisms that possess this *nosZII* gene also harbor a highly truncated version of the denitrification pathway without any nitrite reductase or N_2_O-producing nitric oxide reductase, and, therefore, can only consume N_2_O (Graf et al., 2014). The abundance and phylogenetic diversity of *nosZII* microorganisms was found to mediate the soil N_2_O sink capacity in European soils (Jones et al., 2014), showing the importance of understanding the ecology of this microbial guild for mitigating N_2_O emissions.

Agriculture accounts for about 60% of all N_2_O emissions from global anthropogenic sources (Syakila and Kroeze, 2011). A compilation of more than 1215 measurements of N_2_O emissions from agricultural and natural soils showed that agricultural practices, such as the N application rate, type of crop and type of fertilizer, affected the emissions (Stehfest and Bouwman, 2006). More recently, Shcherbak et al. (2014) reported that the response of soil N_2_O emissions to nitrogen fertilizer was nonlinear for synthetic fertilizers and most crop types. The effect of agricultural practices has often been described in terms of changes in soil substrates or environmental conditions, which can also affect soil microbial communities in various ways. For example, soil amendment with peat stimulated the relative abundance of the Alphaproteobacteria, but reduced the relative abundance of Firmicutes (Wessen et al., 2010). Organic farming increased richness, decreased evenness and shifted the structure of the soil microbial community when compared with conventionally managed soils amended with mineral fertilizers (Hartmann et al., 2014) Shifts in abundance and structure of the denitrifier community have also been reported in response to the fertilization regime (Hallin et al., 2009; Clark et al., 2012; Tatti et al., 2014) or in response to land use intensity (Meyer et al., 2013). However, very little is known about how soil management could affect microorganisms belonging to the newly described *nosZII* clade while agricultural practices that foster these microorganisms are of interest for mitigating N_2_O emissions.

This study was therefore set out to determine how the *nosZI* and *nosZII* N_2_O-reducing communities responded to various agricultural practices in two different arable farming systems. It also assessed the relationships between diversity, composition, and abundance of the N_2_O-reducing microbial communities and N-gas production (N_2_O and N_2_) by denitrification. The study was based on two randomized block experiments localized at the same site, one with an annual rotation with 5 different management practices (ORE) and one with a perennial crop system with 4 different management practices (BE).

## Results

### Potential N_2_O production, potential denitrification activity (N_2_O+N_2_) and denitrification end-product ratio [rN_2_O/r(N_2_O+N_2_)]

**Figure 1:**
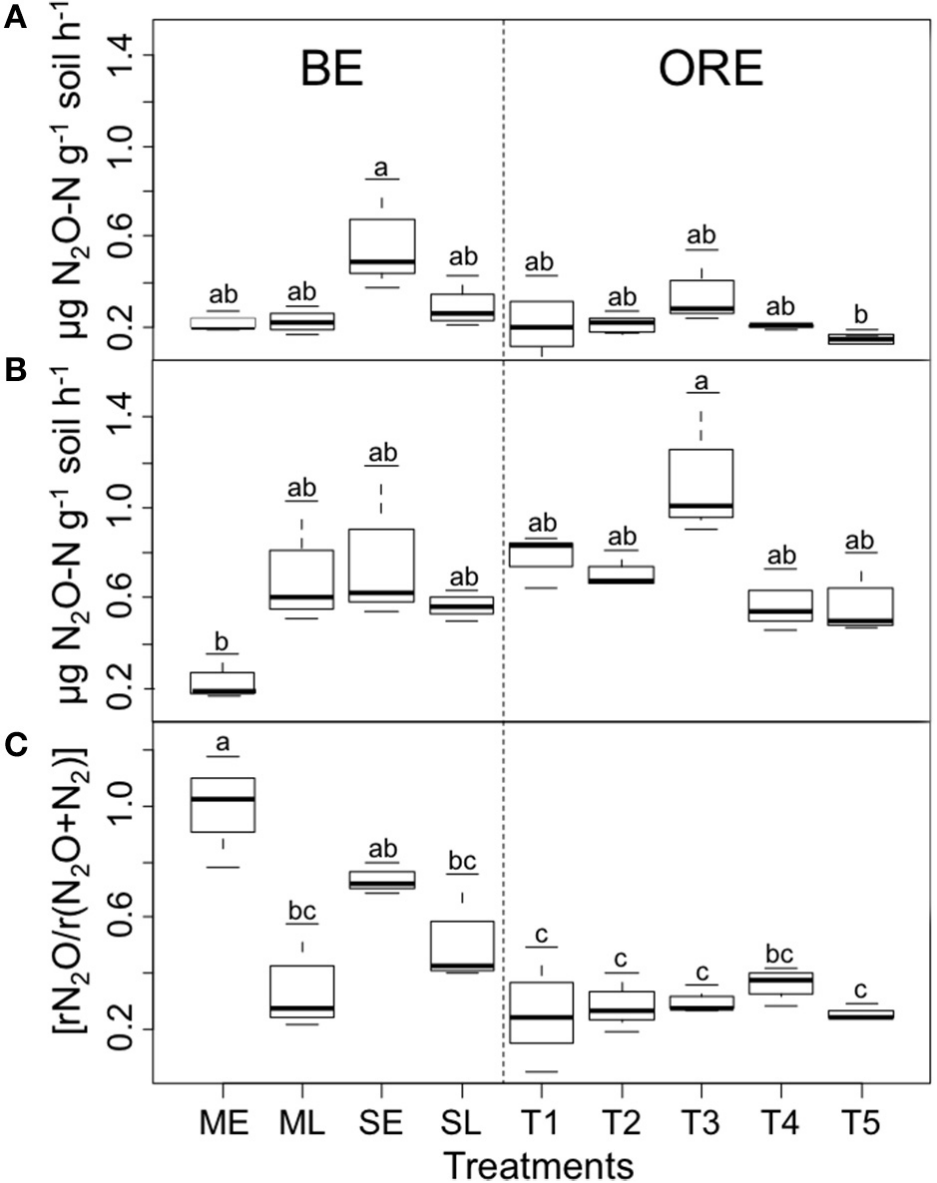
**Potential N_2_O emissions (A), potential denitrification activity (B) and denitrification gaseous end-product ratio [rN_2_O/r(N_2_O+N_2_)] (C)**. Means ± sem per treatments within each experimental block are given. BE and ORE represent the respective cropping systems. Significant differences between treatments are indicated with different letters (anova followed by Tukey HSD test, *P* < 0.05).

To assess the activity of the N_2_O reducing microbial communities, the potential N_2_O production and potential denitrification activity (PDA) were quantified and used to calculate the denitrification end-product ratio [rN_2_O/r(N_2_O+N_2_)]. The potential activity of denitrifying microorganisms varied in all cropping systems, ranging from 0.03 (CI_95%_ = [0− 0.09]) to 0.85 (CI_95%_ = [0.79−0.91]) and 0.17 (CI_95%_ = [0.07−0.27]) to 1.51 (CI_95%_ = [1.41−1.61]) μg N_2_O-N g^−1^ soil DW h^−1^ for potential N_2_O and PDA, respectively (Figures 1A,B). The [rN_2_O/r(N_2_O+N_2_)] ratio ranged between 0.18 (CI_95%_ = [0.08–0.28]) and 1 (CI_95%_ = [0.9–1.1]) (Figure 1C) and was significantly higher (*P* < 0.001) for BE than for ORE cropping system with an average of 0.65 (CI_95%_ = 0.81–0.48) and 0.29 (CI_95%_ = 0.24–0.34), respectively. There were significant differences in [rN_2_O/r(N_2_O+N_2_)] between the early (ME) and late harvest (ML) practices for plots planted with *Miscanthus giganteus* (*P* < 0.05) (Figure 1B) with the denitrification end product being mainly N_2_O in the early harvested plots with a [rN_2_O/r(N_2_O+N_2_)] close to 1 (Figure 1C). For switchgrass, there was the same tendency to have a higher [rN_2_O/r(N_2_O+N_2_)] with the early harvest practice, although this was not significant.

### Abundance of total bacteria, N_2_O-producers and N_2_O-reducers

The genes encoding catalytic enzymes involved in N_2_O production (*nirK* and *nirS*) and N_2_O reduction (*nosZI* and *nosZII*) were quantified by Real-Time quantitative PCR (qPCR) and used as proxies for the abundances of the corresponding functional communities. The 16S rRNA gene copy number, which was used to estimate the abundance of the total bacteria community, varied from 1.3 × 10^9^ to 5.1 × 10^9^ copy numbers g-1 oil DW without any significant difference between treatments (data not shown). The relative abundances of *nirS* and *nirK* communities were similar, ranging from 2.5 to 7.6% of the total bacterial community (Figure S1). The *nosZI* community was significantly (*P* < 0.05) more abundant than the *nosZII* community, ranging from 4.9 to 8.5% and 0.28 to 2.9% of the total bacteria, respectively (Figure S1). No significant differences in N_2_O-producers and N_2_O-reducers abundances were found between treatments (Figure S1). However, *nosZII* abundance was higher in BE than in ORE (*P* < 0.01).

### N_2_O-reducer diversity

**Figure 2:**
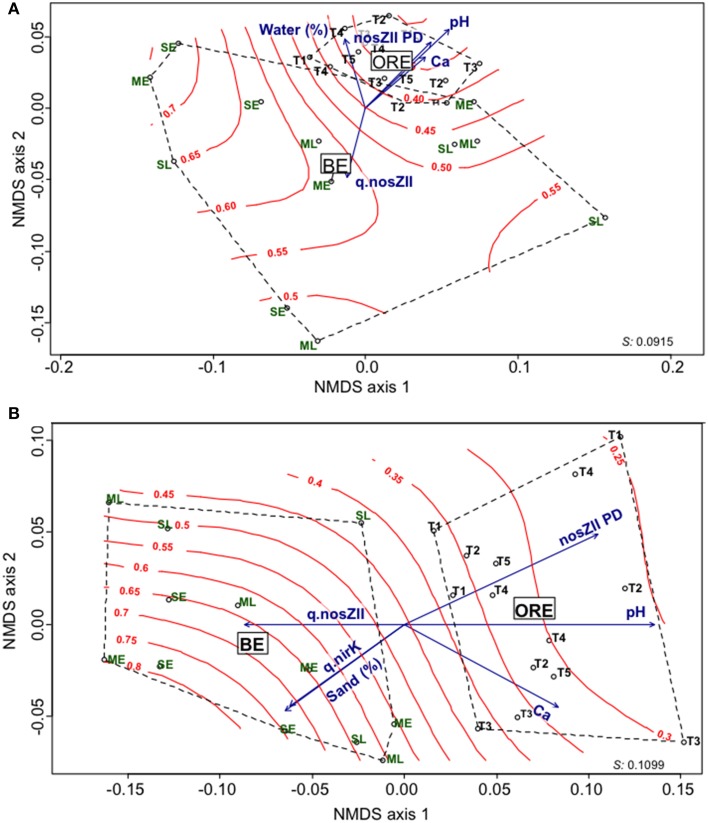
**NMDS ordinations of ***nosZ*** weighted unifrac distance matrices**. **(A)** Variation in *nosZI* community structure. **(B)** Variation in *nosZII* community structure. Red curves represent the final denitrification product [rN_2_O/r(N_2_O/N_2_)]. Significant explanatory variables are represented as blue vectors (*P* < 0.05), Ca (calcium g kg^−1^ dw soil), q.nosZII and q.nirK correspond to the quantification by qPCR of nitrous oxide reductase community (clade II) and copper nitrite reductase community, respectively (copy number g^−1^ dw soil); sand and water content are expressed in percentage. The lengths of the arrows are proportional to the strength of the correlation. Stress values are indicated at the bottom right of each panel.

To assess whether the agricultural practices could drive the composition and structure of the N_2_O-reducer community, the diversity of both *nosZI* and *nosZII* clades was characterized by 454 pyrosequencing. 123,130 *nosZI* and 121,500 *nosZII* sequences were obtained from samples after quality filtering. Similarity-based clustering of sequences gave an average of 162 (CI_95%_ = 133–191) and 312 (CI_95%_ = 279–345) OTUs for *nosZI* and *nosZII* respectively in BE, and 158 (CI_95%_ = 136–180) and 355 (CI_95%_ = 330–380) OTUs for *nosZI* and *nosZII* respectively in ORE. In both BE and ORE, the *nosZII* community had a significantly higher richness than *nosZI* (*P* < 0.0001). Within BE and ORE, the agricultural practices had no significant effect on the α-diversity of the N_2_O-reducing communities (Table S1). An analysis of *nosZI* and *nosZII* phylogenies showed that the most abundant sequences in ORE and BE were similar (Figures S2, S3), with *nosZII* sequences affiliated mainly to *nos*Z from Bacteroidetes while *nosZI* sequences were affiliated to *nos*Z from Alphaproteobacteria and Betaproteobacteria. Further examination of the β-diversity by non-metric multidimensional scaling (NMDS) and analysis of similarity (ANOSIM) showed (Figures 2A,B) no clustering of samples according to the agricultural practices. However, differences in both *nosZ* communities between annual rotation and perennial cropping systems were significant, but stronger for *nosZII* communities, (*R* = 0.43, *P* < 0.0001 and *R* = 0.77, *P* < 0.0001 for *nosZI* and *nosZII* respectively). Fitting the environmental variables onto the ordination plot showed that pH and calcium were significant explanatory variables (*P*<*0.05*) for the community structure of both guilds. The *nosZI* community structure was also related to the water content while the abundance of nitrite reductase genes (*nirK*) and sand content were related to the *nosZII* community structure (Figure 2B). The [rN_2_O/r(N_2_O+N_2_)] surface was fitted onto the ordination plot and showed a strong relationship between the *nosZII* community structure, its diversity and the denitrification end products with a lower proportion of N_2_O produced when the *nosZII* diversity increased (Figure 2B).

### Denitrification activity and end product ratio as a function of soil properties, abundance and diversity of the N_2_O-reducing community

**Figure 3:**
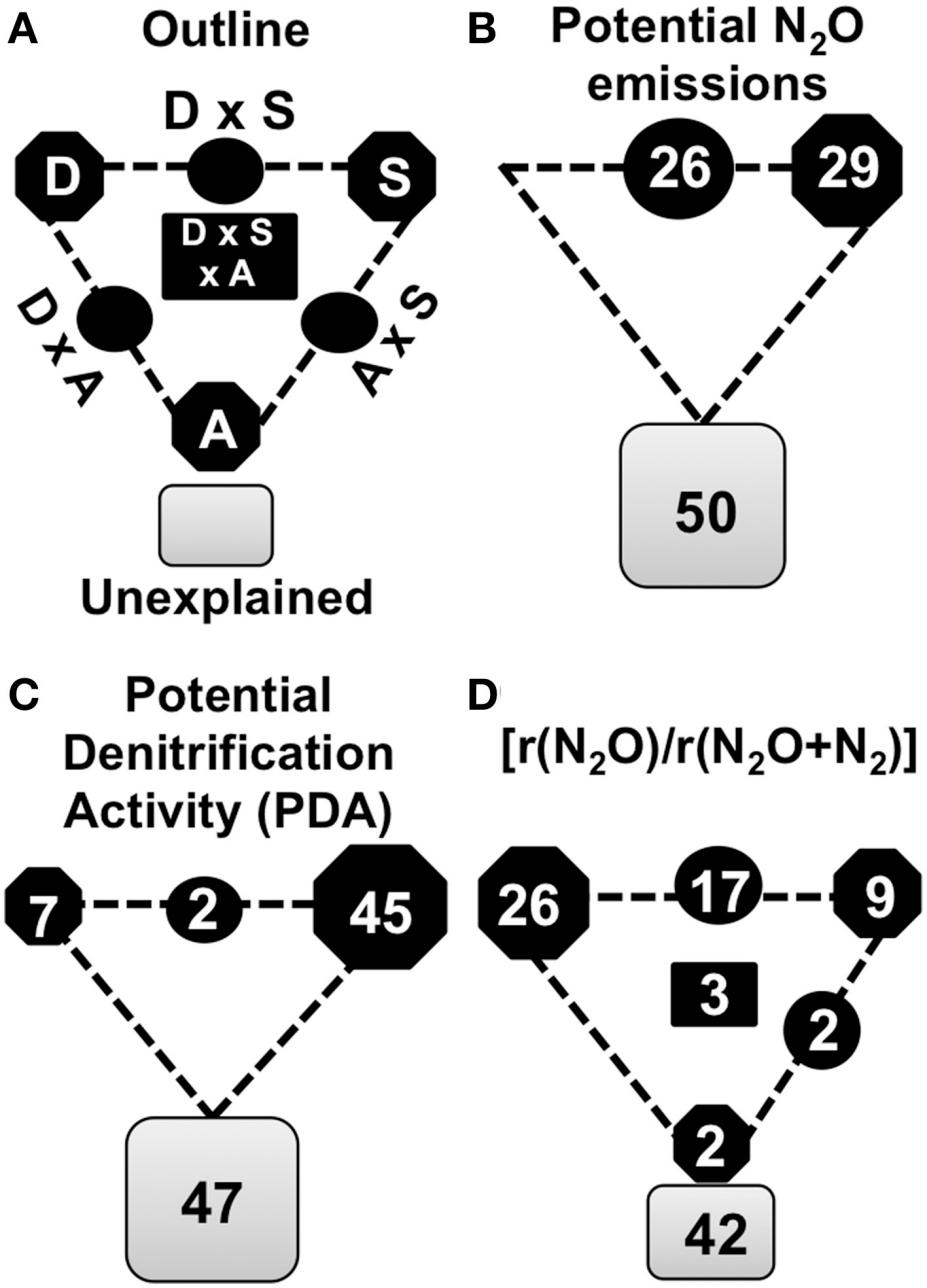
**Variation partitioning of denitrification activities. (A)** Variance in denitrification activities was partitionned into *nosZ* diversity **(D)**, soil physicochemical properties (S), denitrifiers abundance **(A)** and by combinations of predictors. Geometric areas are proportional to the respective percentages of explained variation. The edges of the triangle depict the variation explained by each factor alone. Percentages of variation explained by interactions of two or all factors are indicated on the sides and in the middle of the triangles, respectively. **(B)** Variance partitioning of potential N_2_O emissions. **(C)** Variance partitioning of potential denitrification activity (PDA). **(D)** Variance partitioning of final denitrification product [r(N_2_O/r(N_2_O+N_2_)]. The variables used for each variation partitioning are indicated in the Table S2.

We used variance partitioning technique to quantify the relative contribution of the different groups of variables to the variation in N_2_ production by denitrification across samples (Figures 3A–D). The physical and chemical characteristics of the soil, the abundance of N_2_O producers and reducers, and the diversity of N_2_O reducers were used as explanatory variables. After model selection using multiple linear regressions (Table S2), the physical and chemical properties of the soil were found to be the variables that contributed most to the potential N_2_O production and PDA, explaining up to 29 and 45% of the variance, respectively (Figures 3B,C). In contrast, the [rN_2_O/r(N_2_O+N_2_)] was mostly explained by the diversity of the N_2_O-reducers (26%). Interactions between physical and chemical properties of the soil and the diversity of the N_2_O-reducing communities accounted for 26 and 17% of potential N_2_O production and [rN_2_O/r(N_2_O+N_2_)], respectively (Figures 3B,D). The importance of *nosZII* diversity for the end product ratio of denitrification was also suggested by the strong negative correlation between the (rN_2_O/r(N_2_O+N_2_)) and the *nosZII* diversity (*r* = -0.70, *P* < 0.0001) (Figure S4). The abundance of the communities studied made only a marginal contribution, explaining 2% of the variance in [rN_2_O/r(N_2_O+N_2_)].

### Discussion

The recent identification of a previously unknown clade of N_2_O reducers (Sanford et al., 2012; Jones et al., 2013), whose abundance and phylogenetic diversity are critical for the soil N_2_O sink capacity (Jones et al., 2014), raised the question of whether it would be possible to mitigate N_2_O GHG emissions by selecting agricultural practices that favor these microorganisms. A large body of literature shows that land management can drive microbial communities (Clark et al., 2012; Lauber et al., 2013; Hartmann et al., 2014). Here, our intent was not to compare the impact of individual agricultural practices between the two cropping systems but rather to assess how practices for a particular system, such as tillage, residue management, quantity and nature of N inputs in ORE and biomass crop species and harvest date in BE, affect N_2_O-reducing communities and N_2_ production. We did not observe any significant effect of the practices for either BE or ORE on either the diversity or the abundance of the N_2_O-reducing communities (Table S1 and Figure S1). In contrast, previous results showed differences in the denitrifier community under different fertilization regimes (Hallin et al., 2009) or tillage systems (Melero et al., 2011). Significant effects of the cropping systems on the total bacterial communities were also reported (Hartmann et al., 2014). One common feature of these studies is that they were based on long term experiments that had been running for up to 50 years. This discrepancy with our results could be due to the fact that the ORE and BE systems were established only in 2010 and 2006, respectively, which has not allowed a strong differentiation in soil properties between practices within a cropping system (Table 1). On the other hand, the comparison of ORE and BE did show differences in the N_2_O reducing communities with a higher *nosZII* abundance for ORE than for BE (*P* < 0.01). *NosZII* community richness estimated as OTU numbers was also significantly higher in ORE than in BE (Table S1). Furthermore, NMDS also showed significant clustering according to perennial and annual rotation cropping systems for *nosZII* and to a lesser extent for *nosZI* (Figures 2A,B). Since it is not possible to distinguish the effect of practices from soil legacy or age effects between ORE and BE, it cannot be concluded that the observed changes in the *nosZII* community were due to differences in agricultural practices. However, the stronger response of the *nosZII* community structure and abundance indicates that this recently identified clade is more sensitive to environmental changes than the *nosZI* clade and is therefore more likely to be driven by the land use type.

**Table 1:** **Description of treatments and soil physicochemical properties**.

**Treatment**	**Cropping system**	**Crops**	**Management practices^1^**	**N input^2^**	**% Water**	**pH**	**% Clay^3^**	**% Loam^3^**	**% Sand^3^**	**Total C^4^**	**Total N^4^**	**OM^4^**	**Ca^4^**	**CEC^5, 6^**
ML	BE	Miscanthus (*M. giganteus*)	Late harvest	120	21.7^c^ ± 0.9	7.2^ab^ ± 0.5	20.2 ± 3.6	74.0 ± 3.3	5.6 ± 0.4	11.5 ± 1.0	1.0 ± 6.10^−2^	19.9 ± 1.9	1.3 ± 0.6	11.1 ± 2.9
ME	BE	Miscanthus (*M. giganteus*)	Early harvest	120	23.0^bc^ ± 0.1	7.4^ab^ ± 0.6	20.9 ± 1.7	73.5 ± 2.4	6.0 ± 1.3	10.9 ± 0.6	1.0 ± 4.10^−2^	18.9 ± 1.0	2.3 ± 2.6	11.6 ± 1.0
SL	BE	Switchgrass (*P. virgatum*)	Late harvest	120	23.3^ab^ ± 1.1	7.4^ab^ ± 0.5	21.9 ± 2.4	72.3 ± 1.3	5.7 ± 1.1	11.8 ± 0.8	1.1 ± 6.10^−2^	20.5 ± 1.3	1.3 ± 0.6	12.3 ± 1.1
SE	BE	Switchgrass (*P. virgatum*)	Late harvest	120	24.7^abc^ ± 0.6	6.8^b^ ± 0.1	20.1 ± 3.3	73.8 ± 2.8	6.0 ± 0.5	12.0 ± 0.4	1.1 ± 5.10^−2^	20.7 ± 0.8	1.0 ± 0.0	10.8 ± 1.8
T1	ORE	Peas, rapeseed, wheat, barley, corn, wheat	Conventional tillage, straw addition, high N input, non-legume intercrop	100	25.1^ab^ ± 0.9	7.9^a^ ± 0.2	21.1 ± 4.0	74.1 ± 3.4	4.5 ± 0.5	10.7 ± 0.8	1.0 ± 4.10^−2^	18.5 ± 1.4	3.0 ± 2.9	12.3 ± 1.7
T2	ORE	Peas, rapeseed, wheat, barley, corn, wheat	Reduced tillage, straw addition, high N input, non-legume intercrop	100	24.8^ab^ ± 0.5	7.9^a^ ± 0.3	17.7 ± 0.5	77.0 ± 0.06	4.9 ± 0.4	10.8 ± 0.2	1.0 ± 1.10^−2^	18.6 ± 0.5	3.7 ± 2.8	10.5 ± 0.5
T3	ORE	Peas, rapeseed, wheat, barley, corn, wheat	Reduced tillage, straw removal, high N input, non-legume intercrop	100	24.4^ab^ ± 1.0	7.9^a^ ± 0.2	20.5 ± 2.8	74.4 ± 3.2	4.7 ± 0.7	11.4 ± 0.7	1.1 ± 6.10^−2^	19.7 ± 1.3	4.0 ± 5.8	12.1 ± 1.5
T4	ORE	Peas, rapeseed, wheat, barley, corn, wheat	Conventional tillage, straw addition, low N input, non-legume intercrop	40	25.2^a^ ± 1.1	8.0^a^ ± 0.2	19.8 ± 3.3	75.1 ± 3.3	4.8 ± 0.2	10.7 ± 0.4	1.0 ± 5.10^−2^	18.6 ± 0.8	3.3 ± 2.8	11.7 ± 1.9
T5	ORE	Peas, rapeseed, wheat, barley, corn, wheat	Conventional tillage, straw addition, low N input, legume intercrop	40	25.0^ab^ ± 0.4	8.1^a^ ± 0.1	17.8 ± 1.6	77.1 ± 1.2	4.8 ± 0.5	10.7 ± 0.3	1.0 ± 5.10^−2^	18.4 ± 0.6	3.4 ± 1.7	10.4 ± 1.0

*Means and 95% confidence interval of soil physicochemical properties are given per treatment within each experimental block. Significant differences between treatments are indicated with different letters: “a”, “b”, and “c” (anova followed by Tukey HSD test, P < 0.05)*.

*^1^“Late” and “early” harvest correspond to February and October, respectively, “conventional tillage” is applied to a 20 cm depth while “reduced tillage” to 6 cm, “intercrop” is a non-leguminous crop for all treatments at ORE except for T5; ^2^N input (Kg N ha^−1^ y^−1^); ^3^Size fractions based on μm Atterberg scale; Clay = 2 μm, Loam = 2-50 μm, and Sand = 50-2000 μm; ^4^g kg^−1^; ^5^cation exchange capacity; ^6^cmol kg^−1^*.

The agricultural practices studied also had little impact on PDA and N_2_O production. However, larger differences were observed when calculating the denitrification gaseous end-product ratio (rN_2_O/r(N_2_O+N_2_)) with, in particular, early harvest of *M. giganteus* giving a significantly higher proportion of N_2_O than late harvest. Previous studies at BE showed that late harvest of *M. giganteus* gave a significant net input and accumulation of organic matter due to senescence and leaf fall between early and late harvest (Amougou et al., 2011, 2012; Cadoux et al., 2014). This type of mulch is known to improve soil moisture by reducing soil surface evaporation, resulting in a lower partial pressure of oxygen (pO_2_)_._ This is consistent with our results since previous studies have shown that production of N_2_O relative to N_2_ during denitrification in soils is strongly influenced by carbon availability and pO_2_ (Firestone et al., 1980; Murray and Knowles, 2004; Walker et al., 2008; Giles et al., 2012; Saggar et al., 2013) with, for example decreasing proportion of N_2_O produced during denitrification with decreasing O_2_ concentrations (Firestone et al., 1980). However, the harvesting date had no significant effect on the denitrification gaseous end-product ratio for switchgrass. This might be due to the fact there is no leaf deposition between late and early harvest for switchgrass. Similarly, to the N_2_O reducer community structure, significant differences in the N_2_O ratio were observed between the BE and ORE cropping systems with a lower end-product ratio in ORE (*P* < 0.001). In agreement with previous results (Jones et al., 2014), a strong negative correlation was found between the (rN_2_O/r(N_2_O+N_2_)) ratio and *nosZII* diversity (*r* = -0.70, *P* < 0.0001) (Figure S4), highlighting the importance of *nosZII* N_2_O reducers in the N_2_O-reducing capacity of a soil.

We found that the abundance of *nosZII* N_2_O reducers was significantly correlated with several soil properties such as pH, Ca concentration, soil moisture and total N. This was not the case for *nosZI* which was correlated solely with the C:N ratio, confirming that the *nosZII* clade is more sensitive to changes in environmental conditions than the *nosZI* clade. The analysis of the community structure showed that two N_2_O-reducer clades shared a certain number of explanatory variables but there were also distinct explanatory variables for each clade. The structure of both communities was driven by pH and calcium, while water content was related to clade I and sand to clade II (Figures 3A,B). To our knowledge, the difference in the response of the two N_2_O reducer clades to soil properties has only been assessed in one previous study (Jones et al., 2014). Using structural equation modeling, Jones et al. (2014) showed that soil texture was a more important driver of the abundance of the *nosZI* community whereas soil pH affected the abundance of the *nosZII* community only. Overall, our results, which showed that different factors influenced the *nosZI* and *nosZII* clades, confirm niche partitioning between the two N_2_O reducing communities. They also indicate that agricultural practices could affect *nosZI* and *nosZII* communities in different ways with consequences for N_2_O reduction.

Variation partitioning analysis was applied to disentangle the contribution of soil properties, the abundance of N_2_O producers and reducers, and the diversity of N_2_O reducing microbial communities to the production of N_2_O and N_2_ as the denitrification end products. Potential N_2_O production (29%) and PDA (45%) were mostly explained by soil properties. Water content, C/N ratio, Cation Exchange Capacity (CEC) and pH were the major soil properties explaining N_2_O production, while sand content, total nitrogen and the C/N ratio explained PDA. Accordingly, nitrate and carbon availability, pO_2_ and related variables such as water content and soil texture are well known proximal factors regulating N_2_O and PDA rates (Baggs, 2011; Giles et al., 2012). pH is also known to be a master regulator of biological processes in soils (Enwall et al., 2005; Giles et al., 2012; Petersen et al., 2012; Saggar et al., 2013; Liu et al., 2014) including the reduction of N_2_O into N_2_ by nitrous oxide reductase (Firestone et al., 1980). Unlike other studies (Hallin et al., 2009; Petersen et al., 2012; Jones et al., 2014), the abundance of the N_2_O producing and reducing communities did not explain variations in PDA nor in potential N_2_O production (Figures 3B,C). According to Petersen et al. (2012) the abundance of denitrifiers was correlated with PDA only when large variations in fluxes were observed, which was not the case in this study. In contrast to potential N_2_O production and PDA, the variance in the proportion of N_2_O emitted by denitrification [rN_2_O/r(N_2_O+N_2_)] was mostly explained by the diversity of the *nosZII* clade (26%), and the interaction between this diversity and soil properties (17%) (Figure 3D). This is in agreement with previous research showing a positive correlation between the ability of the soil to consume N_2_O and the phylogenetic diversity of the *nosZII* clade (Jones et al., 2014). It also illustrates the importance of the *nosZII* community in determining the nature of the denitrification gaseous end-products through their capacity to act as a N_2_O sink.

Overall, our results showed that the newly described *nosZII* clade is the strongest predictor of the [rN_2_O/r(N_2_O+N_2_)] ratio while soil properties are the main drivers of potential denitrification and N_2_O production. They also showed that the two clades of N_2_O reducers were not affected by the same soil properties, suggesting niche partitioning. The *nosZII* clade was more sensitive to environmental changes than the *nosZI* clade, which may make it easier to foster this group using agricultural practices as a new strategy for mitigating N_2_O emissions. Further studies are required to determine the effect of different agricultural practices on the abundance and diversity of the *nosZII* clade, in sites with different pedoclimatic conditions to provide more information on the ecology of this recently described functional guild. Moreover, due to the increasing evidence that fungi can produce N_2_O and N_2_ by denitrification and co-denitrification (Laughlin and Stevens, 2002; Wei et al., 2014; Maeda et al., 2015), respectively, the response of these microorganisms to agricultural practices should also be considered to circumvent any tradeoff.

## Materials and methods

### Experimental design and sampling

Soil samples were collected in October 2013 from two randomized field experiments ORE (49°52′25.615″N, 3°1′53.914″E) and BE (49°52′19.29″N, 3°0′47.267″E) located at the same site near Estrées-Mons, France, which has both annual rotation and perennial crop systems, as well as various agricultural practices. A description of the practices can be found in Table 1. Replicated plots with the same practices were randomly distributed within each block experiment. Briefly, the ORE experiment, which consisted in 5 treatments (T1–T5), was set up in 2010 to study the effect of soil tillage, crop residue management, fertilization rate and substitution of mineral N input by fixation by legumes on biogeochemical cycles and soil biodiversity (www.soere-acbb.com). The BE experiment was set up in 2006 to compare the productivity and environmental impacts of various energy crop systems including perennial crops such as *Miscanthus giganteus* and *Panicum virgatum* and differences in management practices such as early or late harvest (ML, ME, SL, and SE). Three replicate samples were collected for each combination of cropping system and management practices, each being a composite sample of five subsamples (soil cores of 2.5 cm by 20 cm) from each plot. Samples were frozen (−20°C) until further analysis. The physical and chemical soil characteristics were measured for all samples (INRA Laboratory of Soil Analysis, Arras, France) (Table 1).

### Potential denitrification activity (PDA) and potential N_2_O production

Potential denitrification activity (N_2_O + N_2_) and potential nitrous oxide production (N_2_O) were measured using the acetylene inhibition technique (Yoshinari et al., 1977). For each sample 10 g of fresh weight soil was wetted with 20 ml of distilled water and was amended with a final concentration of 3 mM KNO_3_, 1.5 mM succinate, 1 mM glucose, and 3 mM acetate. To determine the potential denitrification activity, acetylene was added to reach 0.1 atm partial pressure followed by 30 min incubation at 25°C and agitation (175 rpm). Gas samples were taken every 30 min for 150 min (Pell et al., 1996). The N_2_O concentrations were determined using a gas chromatograph (Trace GC Ultra, Thermo Scientific) equipped with an EC-detector.

### Nucleic acid extraction and abundance of bacterial communities

DNA extraction for all samples was performed in accordance with ISO 11063 (Petric et al., 2011). 0.25 g of soil was homogenized with a 1 ml homogenization buffer for 30 s at 1600 rpm in a mini-bead beater cell disruptor (Mikro-Dismembrator S; B Braun Biotech International), followed by centrifugation at 14000 × g for 1 min to eliminate soil and cell debris. For protein precipitation, supernatant was incubated on ice for 10 min with 1/10 volume of 3M sodium acetate and centrifuged (14000 × g, 5 min, 4°C). The DNA was precipitated by adding one volume of cold isopropanol (−20°C) over 24 h. The mix was then centrifuged for 30 min at 14,000 g (4°C), the resulting pellet was washed with 70% ethanol, and the DNA was resuspended with 100 μL of TE buffer (pH 8). The DNA was purified in two steps: first using polyvinylpolypyrrolidone (PVPP) microbiospin columns (Bio-Rad, CA, USA), and then a Sepharose 4G column (Sigma-Aldrich, United Kingdom). The DNA quality was checked by electrophoresis on agarose gel and quantified by spectrofluorometer using the Quant-iT PicoGreen® dsDNA Assay Kit (Invitrogen, Cergy-Pontoise, France) following the manufacturer's instructions.

The abundance of denitrifiers was assessed by real-time quantitative PCR (qPCR) by targeting N_2_O-producers, *nirK* and *nirS* (Henry et al., 2004; Kandeler et al., 2006) and N_2_O-reducers *nosZI* (Henry et al., 2006; Jones et al., 2013) and *nosZII* (Table S3). Abundance of total bacteria was assed using 16S rRNA primers (Muyzer et al., 1993) as previously described (López-Gutiérrez et al., 2004). qPCR Reactions were carried out in a StepOnePlus Real time PCR System (Life Technologies, Carlsbad, CA, USA). The abundance was based on the increasing fluorescence intensity of the SYBR Green dye during amplification. The qPCR assay was carried out in a 15 μl reaction volume containing 1 ng of DNA, 7.5 μl of SYBRgreen PCR Master Mix (Absolute qPCR SYBR GreenRox, Thermo, Courtaboeuf, France), 1 μM of each primer, 250 ng of T4 gene 32 (QBiogene, Illkrich, France). Before assessing the abundance of the bacterial communities, an inhibition test was performed by mixing DNA extracts with a known amount of control plasmid DNA and no inhibition was detected. Three independent quantitative qPCR assays were performed for each gene. Controls and no-template controls giving null or negligible values were run for each quantitative qPCR assay. The qPCR efficiencies for the various genes ranged between 70 and 96%.

### Phylogenetic diversity of N_2_O-reducers

A diversity analysis of *nosZI* and *nosZII* was performed by 454 pyrosequencing as previously described in Jones et al. (2014). Briefly, the DNA was prepared using a two-step PCR procedure (Berry et al., 2012). In the first step, 20 PCR cycles were performed with primers *nosZI* and *nosZII* (Table S4) in a 25 μl reaction volume containing 5 μl 5 × Taq Buffer (GoTaq, Promega, Madison, U.S.A.), 2 μM of each primer, 250 ng of T4 gene 32 (QBiogene, Illkrich, France), 0.125 μl of DNA Polymerase (GoTaq, Promega, Madison, U.S.A.), 200 μM(each) deoxyribonucleoside triphosphate, and 1 ng of template DNA. In the second step, 4 μL of the PCR products of the first reaction were amplified in a 50 μl reaction volume containing 10 μl 5 × Taq Buffer (GoTaq, Promega, Madison, U.S.A.), 200 μM (each) deoxyribonucleoside triphosphate, 1 μM of each primer, 0.25 μl of DNA Polymerase (GoTaq G2, Promega, Madison, U.S.A.). In the second PCR 15 or 18 cycles PCR were performed using the forward primers preceded by 10 bp-long barcodes, the sequencing key and the forward sequencing adapter; the reverse primers being only preceded by the sequencing key and the reverse sequencing adapter as described in Jones et al. (2014) (Table S4). Because there were only very small amounts of products for *nosZII* after the first PCR, the second PCR for this gene was extended to 18 cycles. The product of 3 independent second PCR was then gel extracted and purified using the QIAEX II kit (Qiagen; France). Pyrosequencing was performed by Genoscreen (Lille, France) on a Roche's 454 FLX Genome Sequencer according to manufacturer's instructions.

### Sequence processing

The QIIME pipeline (Caporaso et al., 2010a) was used for quality trimming of raw 454 pyrosequencing data (QIIME version 1.8.0). The minimum and maximum sequence lengths were 230 and 410 bp respectively. Sequences with an average score below 25 using a sliding window of 50 bp were discarded. After quality checking, 123,130 sequences were found for *nosZI* and 121,500 sequences for *nosZII*. Sequences were then processed using the “pick_otus.py” script within QIIME, and the “usearch” option (Edgar, 2010) with reference-based and *de novo* chimera checking, and clustering of sequences at 97% similarity. Raw sequences were deposited at the NCBI under the accession number SRP058080. The process of raw sequence submission was greatly simplified by using the *make.sra* command of Mothur software (Schloss et al., 2009).

### *nosZ* phylogeny

Reference sequences for *nosZ* were downloaded from all 4135 draft and completed microbial genome nucleotide sequences available in the National Center for Biology Information (NCBI) (Jones et al., 2014). These reference sequences were used as templates for aligning 454 reads with PYNAST (Caporaso et al., 2010b). Phylogenetic trees for *nosZI* and *nosZII* were constructed with fastree (Price et al., 2010) and ITOL was used to visualize and manipulate of the trees (Letunic and Bork, 2007).

### Statistical analysis

Statistical analysis and graphics were produced using the R statistical software, R version 3.0.3, (R Core Team, 2013) and the *agricolae* (Mendiburu, 2014) and *vegan* (Oksanen et al., 2015) packages. The effect of agricultural practices and cropping systems was determined by analysis of variance and *post hoc* Tukey HSD test. Collinearity between explaining variables within each group (soil properties, microbial community abundances, and denitrifiers diversity) was checked, and one of each pair of collinear variables was kept for subsequent analyses. Non-metric MultiDimensional Scaling (NMDS) of the Unifrac distance matrices (unweighted and weighted) was used to describe community structure. Ordinations with the lowest stress values were used. The soil properties, community abundances and diversity were plotted onto the ordination map as vectors. Permutation tests (*n* = 10000) were used to test the significance of vector fits and only significant ones were depicted (*P* < 0.05). Vector and surface fitting of variables within ordinations were performed using the *envfit* and *ordisurf* functions in the vegan package respectively. ANalysis Of SIMilarity (ANOSIM) was used to test for significant differences in community structure between cropping systems (permutations = 1999, *P* < 0.05).

Significant explanatory variables of [rN_2_O/r(N_2_O+N_2_)], potential N_2_O and PDA were chosen by linear regression and model selection (backward) and by minimizing the Akaike Information Criterion (AIC). The statistical significance was assessed by 1000 permutations of the reduced model. The resulting significant explanatory variables (Table S2) were used to access their contribution to explaining the variation of potential N_2_O, PDA and [rN_2_O/r(N_2_O+N_2_)], using the function *varpart* (Peres-Neto et al., 2006).

### Conflict of interest statement

The authors declare that the research was conducted in the absence of any commercial or financial relationships that could be construed as a potential conflict of interest.
