# Supplementary material for: The diversity of the N2O reducers matters for the N2O:N2 denitrification end-product ratio across an annual and a perennial cropping system
Source: Front Microbiol. 2015 Sep 24;6:971. doi: 10.3389/fmicb.2015.00971 (PMC4585238; doi:10.3389/fmicb.2015.00971)

***Supplementary Material***

**The diversity of the N_2_O reducers matters for the N_2_O:N_2_ denitrification end-product ratio across an annual and a perennial cropping system.**

**L. A. Domeignoz-Horta, A. Spor, D. Bru, M.C. Breuil, F. Bizouard, J. Léonard, L. Philippot***

***Correspondence:** Corresponding Author: Laurent.Philippot@dijon.inra.fr

1. **Supplementary Data**
2. **Supplementary Tables and Figures**

## Table S1. Diversity of the two *nosZ* clades. Means and 95% confidence interval of diversity indices for both *nosZ* clades are given per treatment within each experimental block. Significant differences between treatments are indicated with different letters (anova followed by Tukey HSD test, P<0.05).

|  | *nosZI* | | | | | | *nosZII* | | | | |
| --- | --- | --- | --- | --- | --- | --- | --- | --- | --- | --- | --- |
| Treatment | OTUs | PD | chao1 | Simpson reciprocal | Shannon |  | OTUs | PD | chao1 | Simpson reciprocal | Shannon |
| ML | 167 | 9 | 178 | 24 | 5 |  | 322^bc^ | 23^abc^ | 464^ab^ | 40^c^ | 6^c^ |
|  | ± 21.3 | ± 0.5 | ± 19.7 | ± 14.8 | ± 0.5 |  | ± 23.7 | ± 2.7 | ± 57.8 | ± 21.9 | ± 4.10^-1^ |
| ME | 181 | 11 | 227 | 30 | 6 |  | 318^bc^ | 21^bc^ | 472^ab^ | 33^c^ | 6^c^ |
|  | ± 44 | ± 2.4 | ± 94.7 | ± 7.8 | ± 4.10^-1^ |  | ± 37.6 | ± 2.9 | ± 94.4 | ± 11.3 | ± 3.10^-1^ |
| SL | 163 | 9 | 196 | 24 | 5 |  | 315^bc^ | 23^abc^ | 461^ab^ | 52^bc^ | 7^bc^ |
|  | ± 9 | ± 0.4 | ± 23.8 | ± 6.6 | ± 1.10^-1^ |  | ± 49.2 | ± 6.6 | ±159.5 | ± 21.4 | ± 4.10^-1^ |
| SE | 138 | 8 | 165 | 21 | 5 |  | 294^c^ | 20^c^ | 457^b^ | 34^c^ | 6^c^ |
|  | ± 19 | ± 1.2 | ± 25.1 | ± 13.0 | ± 4.10^-1^ |  | ± 31.1 | ± 2.05 | ± 67.7 | ± 5.2 | ± 2.10^-1^ |
| T1 | 161 | 9 | 191 | 14 | 5 |  | 373^ab^ | 29^ab^ | 558^ab^ | 92^ab^ | 7^ab^ |
|  | ± 30 | ± 0.6 | ± 38.1 | ± 5.0 | ± 4.10^-1^ |  | ± 26.2 | ± 1.6 | ± 56.8 | ± 10.4 | ± 6.10^-2^ |
| T2 | 161 | 9 | 196 | 18 | 5 |  | 382^ab^ | 31^a^ | 547^ab^ | 98^a^ | 7^ab^ |
|  | ± 4 | ± 0.4 | ± 23.9 | ± 1.6 | ± 7.10^-2^ |  | ± 29.0 | ± 3.8 | ± 38.1 | ± 16.1 | ± 1.10^-1^ |
| T3 | 176 | 10 | 203 | 16 | 5 |  | 368^abc^ | 28^abc^ | 526^ab^ | 84^ab^ | 7^ab^ |
|  | ± 12.7 | ± 1.1 | ± 18.3 | ± 1.4 | ± 9.10^-2^ |  | ± 24.8 | ± 2.3 | ± 58.6 | ± 18.6 | ± 1.10^-1^ |
| T4 | 175 | 10 | 223 | 18 | 5 |  | 369^abc^ | 27^abc^ | 526 ^ab^ | 93^ab^ | 7^ab^ |
|  | ± 35.1 | ± 1.9 | ± 58.5 | ± 10.1 | ± 5.10^-1^ |  | ± 18.6 | ± 2.2 | ± 21.2 | ± 14.40 | ± 1.10^-1^ |
| T5 | 179 | 10 | 237 | 16 | 5 |  | 404^a^ | 30^a^ | 621^a^ | 110^a^ | 7^a^ |
|  | ± 34.3 | ± 1.1 | ± 74.9 | ± 0.3 | ± 2.10^-1^ |  | ± 14.1 | ± 2.1 | ± 7.1 | ± 20.9 | ± 8.10^-2^ |

**Table S2. Selected explaining variables used for the variation partitioning analyses.** Diversity of *nosZ* clades is represented by Faith’s PD index, water and sand are expressed in %, TN represents total nitrogen (g/kg), and CEC the cation exchange capacity (cmol+/kg). Abundance (nbc/ng DNA) of N_2_O producers and reducers is expressed by q.nirK, q.nirS and q.nosZI, q.nosZII, respectively.

|  | Potential N_2_O | PDA | [rN_2_O/r(N_2_O+N_2_)] |
| --- | --- | --- | --- |
| Diversity nosZ clades | *nosZI*, *nosZII* | *nosZI*, *nosZII* | *nosZII* |
| Soil Physicochemical properties | water, pH, C/N, cec | sand, TN, C/N | sand, C/N |
| Abundance (qPCR) | q.nosZII | - | q.nirK, q.nosZI |

**Table S3. Description of primers used for the qPCR assays.**

| Genes | Primers Name | Sequences | References |
| --- | --- | --- | --- |
| *16S* | Forward:  Reverse: | CCTACGGGAGGCAGCAG  ATTACCGCGGCTGCTGGCA | Muyzer et al., 1993 |
| *nirS* | Forward: nirS4QF  Reverse: nirS6QR | GTSAACGYSAAGGARACSGG  GASTTCGGRTGSGTCTTSAYGAA | Kandeler et al., 2006 |
| *nirK* | Forward: nirK 876F  Reverse: nirK 1040R | CGCRACGGCAASAAGGTSMSSGT  GCCTCGATCAGRTTRTGGTT | Henry et al., 2004 |
| *nosZI** | Forward: nosZ 1840F  Reverse: nosZ 2090R | CGCRACGGCAASAAGGTSMSSGT  CAKRTGCAKSGCRTGGCAGAA | Henry et al., 2006 |
| *nosZII** | Forward: 1153 nosZ 8F  Reverse: 1888 nosZ 29R | CTIGGICCIYTKCAYAC  GCIGARCARAAITCBGTRC | Jones et al., 2013 |

^*^ These same primers were used for the first PCR for 454 pyrosequencing.

**Table S4. Description of primers used for 454 pyrosequencing of *nosZI* and *nosZII*.**

| Sample | Site | Gene | Barcode Name | Primer Sequence |
| --- | --- | --- | --- | --- |
| MPa | BE | *nosZI* | nosZF1-2MID11 | CCA-TCT-CAT-CCC-TGC-GTG-TCT-CCG-ACT-CAG-TGA-TAC-GTC-TCG-CTS-TTY-MT5-GAY-AGY-CAG |
| MPb | BE | *nosZI* | nosZF1-2MID22 | CCA-TCT-CAT-CCC-TGC-GTG-TCT-CCG-ACT-CAG-TAC-GAG-TAT-GCG-CTS-TTY-MT5-GAY-AGY-CAG |
| MPc | BE | *nosZI* | nosZF1-2MID22 | CCA-TCT-CAT-CCC-TGC-GTG-TCT-CCG-ACT-CAG-TAC-GAG-TAT-GCG-CTS-TTY-MT5-GAY-AGY-CAG |
| MTa | BE | *nosZI* | nosZF1-2MID1 | CCA-TCT-CAT-CCC-TGC-GTG-TCT-CCG-ACT-CAG-ACG-AGT-GCG-TCG-CTS-TTY-MT5-GAY-AGY-CAG |
| MTb | BE | *nosZI* | nosZF1-2MID11 | CCA-TCT-CAT-CCC-TGC-GTG-TCT-CCG-ACT-CAG-TGA-TAC-GTC-TCG-CTS-TTY-MT5-GAY-AGY-CAG |
| MTc | BE | *nosZI* | nosZF1-2MID7 | CCA-TCT-CAT-CCC-TGC-GTG-TCT-CCG-ACT-CAG-CGT-GTC-TCT-ACG-CTS-TTY-MT5-GAY-AGY-CAG |
| SPa | BE | *nosZI* | nosZF1-2MID23 | CCA-TCT-CAT-CCC-TGC-GTG-TCT-CCG-ACT-CAG-TAC-TCT-CGT-GCG-CTS-TTY-MT5-GAY-AGY-CAG |
| SPb | BE | *nosZI* | nosZF1-2MID7 | CCA-TCT-CAT-CCC-TGC-GTG-TCT-CCG-ACT-CAG-CGT-GTC-TCT-ACG-CTS-TTY-MT5-GAY-AGY-CAG |
| SPc | BE | *nosZI* | nosZF1-2MID31 | CCA-TCT-CAT-CCC-TGC-GTG-TCT-CCG-ACT-CAG-AGC-GTC-GTC-TCG-CTS-TTY-MT5-GAY-AGY-CAG |
| STa | BE | *nosZI* | nosZF1-2MID33 | CCA-TCT-CAT-CCC-TGC-GTG-TCT-CCG-ACT-CAG-ATA-GAG-TAC-TCG-CTS-TTY-MT5-GAY-AGY-CAG |
| STb | BE | *nosZI* | nosZF1-2MID44 | CCA-TCT-CAT-CCC-TGC-GTG-TCT-CCG-ACT-CAG-TCT-AGC-GAC-TCG-CTS-TTY-MT5-GAY-AGY-CAG |
| STc | BE | *nosZI* | nosZF1-2MID23 | CCA-TCT-CAT-CCC-TGC-GTG-TCT-CCG-ACT-CAG-TAC-TCT-CGT-GCG-CTS-TTY-MT5-GAY-AGY-CAG |
| T1a | ORE | *nosZI* | nosZF1-2MID27 | CCA-TCT-CAT-CCC-TGC-GTG-TCT-CCG-ACT-CAG-ACG-CGA-GTA-TCG-CTS-TTY-MT5-GAY-AGY-CAG |
| T1b | ORE | *nosZI* | nosZF1-2MID33 | CCA-TCT-CAT-CCC-TGC-GTG-TCT-CCG-ACT-CAG-ATA-GAG-TAC-TCG-CTS-TTY-MT5-GAY-AGY-CAG |
| T1c | ORE | *nosZI* | nosZF1-2MID34 | CCA-TCT-CAT-CCC-TGC-GTG-TCT-CCG-ACT-CAG-CAC-GCT-ACG-TCG-CTS-TTY-MT5-GAY-AGY-CAG |
| T2a | ORE | *nosZI* | nosZF1-2MID34 | CCA-TCT-CAT-CCC-TGC-GTG-TCT-CCG-ACT-CAG-CAC-GCT-ACG-TCG-CTS-TTY-MT5-GAY-AGY-CAG |
| T2b | ORE | *nosZI* | nosZF1-2MID35 | CCA-TCT-CAT-CCC-TGC-GTG-TCT-CCG-ACT-CAG-CAG-TAG-ACG-TCG-CTS-TTY-MT5-GAY-AGY-CAG |
| T2c | ORE | *nosZI* | nosZF1-2MID45 | CCA-TCT-CAT-CCC-TGC-GTG-TCT-CCG-ACT-CAG-TCT-ATA-CTA-TCG-CTS-TTY-MT5-GAY-AGY-CAG |
| T3a | ORE | *nosZI* | nosZF1-2MID27 | CCA-TCT-CAT-CCC-TGC-GTG-TCT-CCG-ACT-CAG-ACG-CGA-GTA-TCG-CTS-TTY-MT5-GAY-AGY-CAG |
| T3b | ORE | *nosZI* | nosZF1-2MID1 | CCA-TCT-CAT-CCC-TGC-GTG-TCT-CCG-ACT-CAG-ACG-AGT-GCG-TCG-CTS-TTY-MT5-GAY-AGY-CAG |
| T3c | ORE | *nosZI* | nosZF1-2MID45 | CCA-TCT-CAT-CCC-TGC-GTG-TCT-CCG-ACT-CAG-TCT-ATA-CTA-TCG-CTS-TTY-MT5-GAY-AGY-CAG |
| T4a | ORE | *nosZI* | nosZF1-2MID30 | CCA-TCT-CAT-CCC-TGC-GTG-TCT-CCG-ACT-CAG-AGA-CTA-TAC-TCG-CTS-TTY-MT5-GAY-AGY-CAG |
| T4b | ORE | *nosZI* | nosZF1-2MID44 | CCA-TCT-CAT-CCC-TGC-GTG-TCT-CCG-ACT-CAG-TCT-AGC-GAC-TCG-CTS-TTY-MT5-GAY-AGY-CAG |
| T4c | ORE | *nosZI* | nosZF1-2MID31 | CCA-TCT-CAT-CCC-TGC-GTG-TCT-CCG-ACT-CAG-AGC-GTC-GTC-TCG-CTS-TTY-MT5-GAY-AGY-CAG |
| T5a | ORE | *nosZI* | nosZF1-2MID30 | CCA-TCT-CAT-CCC-TGC-GTG-TCT-CCG-ACT-CAG-AGA-CTA-TAC-TCG-CTS-TTY-MT5-GAY-AGY-CAG |
| T5b | ORE | *nosZI* | nosZF1-2MID37 | CCA-TCT-CAT-CCC-TGC-GTG-TCT-CCG-ACT-CAG-TAC-ACA-CAC-TCG-CTS-TTY-MT5-GAY-AGY-CAG |
| T5c | ORE | *nosZI* | nosZF1-2MID35 | CCA-TCT-CAT-CCC-TGC-GTG-TCT-CCG-ACT-CAG-CAG-TAG-ACG-TCG-CTS-TTY-MT5-GAY-AGY-CAG |
| MPa | BE | *nosZII* | nosZIIF_MID4 | CCA-TCT-CAT-CCC-TGC-GTG-TCT-CCG-ACT-CAG-AGC-ACT-GTA-GCT-5GG-5CC-5YT-KCA-YAC |
| MPb | BE | *nosZII* | nosZIIF_MID5 | CCA-TCT-CAT-CCC-TGC-GTG-TCT-CCG-ACT-CAG-ATC-AGA-CAC-GCT-5GG-5CC-5YT-KCA-YAC |
| MPc | BE | *nosZII* | nosZIIF_MID5 | CCA-TCT-CAT-CCC-TGC-GTG-TCT-CCG-ACT-CAG-ATC-AGA-CAC-GCT-5GG-5CC-5YT-KCA-YAC |
| MTa | BE | *nosZII* | nosZIIF_MID1 | CCA-TCT-CAT-CCC-TGC-GTG-TCT-CCG-ACT-CAG-ACG-AGT-GCG-TCT-5GG-5CC-5YT-KCA-YAC |
| MTb | BE | *nosZII* | nosZIIF_MID4 | CCA-TCT-CAT-CCC-TGC-GTG-TCT-CCG-ACT-CAG-AGC-ACT-GTA-GCT-5GG-5CC-5YT-KCA-YAC |
| MTc | BE | *nosZII* | nosZIIF_MID2 | CCA-TCT-CAT-CCC-TGC-GTG-TCT-CCG-ACT-CAG-ACG-CTC-GAC-ACT-5GG-5CC-5YT-KCA-YAC |
| SPa | BE | *nosZII* | nosZIIF_MID6 | CCA-TCT-CAT-CCC-TGC-GTG-TCT-CCG-ACT-CAG-ATA-TCG-CGA-GCT-5GG-5CC-5YT-KCA-YAC |
| SPb | BE | *nosZII* | nosZIIF_MID2 | CCA-TCT-CAT-CCC-TGC-GTG-TCT-CCG-ACT-CAG-ACG-CTC-GAC-ACT-5GG-5CC-5YT-KCA-YAC |
| SPc | BE | *nosZII* | nosZIIF_MID13 | CCA-TCT-CAT-CCC-TGC-GTG-TCT-CCG-ACT-CAG-CAT-AGT-AGT-GCT-5GG-5CC-5YT-KCA-YAC |
| STa | BE | *nosZII* | nosZIIF_MID15 | CCA-TCT-CAT-CCC-TGC-GTG-TCT-CCG-ACT-CAG-ATA-CGA-CGT-ACT-5GG-5CC-5YT-KCA-YAC |
| STb | BE | *nosZII* | nosZIIF_MID7 | CCA-TCT-CAT-CCC-TGC-GTG-TCT-CCG-ACT-CAG-CGT-GTC-TCT-ACT-5GG-5CC-5YT-KCA-YAC |
| STc | BE | *nosZII* | nosZIIF_MID6 | CCA-TCT-CAT-CCC-TGC-GTG-TCT-CCG-ACT-CAG-ATA-TCG-CGA-GCT-5GG-5CC-5YT-KCA-YAC |
| T1a | ORE | *nosZII* | nosZIIF_MID10 | CCA-TCT-CAT-CCC-TGC-GTG-TCT-CCG-ACT-CAG-TCT-CTA-TGC-GCT-5GG-5CC-5YT-KCA-YAC |
| T1b | ORE | *nosZII* | nosZIIF_MID15 | CCA-TCT-CAT-CCC-TGC-GTG-TCT-CCG-ACT-CAG-ATA-CGA-CGT-ACT-5GG-5CC-5YT-KCA-YAC |
| T1c | ORE | *nosZII* | nosZIIF_MID16 | CCA-TCT-CAT-CCC-TGC-GTG-TCT-CCG-ACT-CAG-TCA-CGT-ACT-ACT-5GG-5CC-5YT-KCA-YAC |
| T2a | ORE | *nosZII* | nosZIIF_MID16 | CCA-TCT-CAT-CCC-TGC-GTG-TCT-CCG-ACT-CAG-TCA-CGT-ACT-ACT-5GG-5CC-5YT-KCA-YAC |
| T2b | ORE | *nosZII* | nosZIIF_MID20 | CCA-TCT-CAT-CCC-TGC-GTG-TCT-CCG-ACT-CAG-ACG-ACT-ACA-GCT-5GG-5CC-5YT-KCA-YAC |
| T2c | ORE | *nosZII* | nosZIIF_MID11 | CCA-TCT-CAT-CCC-TGC-GTG-TCT-CCG-ACT-CAG-TGA-TAC-GTC-TCT-5GG-5CC-5YT-KCA-YAC |
| T3a | ORE | *nosZII* | nosZIIF_MID10 | CCA-TCT-CAT-CCC-TGC-GTG-TCT-CCG-ACT-CAG-TCT-CTA-TGC-GCT-5GG-5CC-5YT-KCA-YAC |
| T3b | ORE | *nosZII* | nosZIIF_MID1 | CCA-TCT-CAT-CCC-TGC-GTG-TCT-CCG-ACT-CAG-ACG-AGT-GCG-TCT-5GG-5CC-5YT-KCA-YAC |
| T3c | ORE | *nosZII* | nosZIIF_MID11 | CCA-TCT-CAT-CCC-TGC-GTG-TCT-CCG-ACT-CAG-TGA-TAC-GTC-TCT-5GG-5CC-5YT-KCA-YAC |
| T4a | ORE | *nosZII* | nosZIIF_MID12 | CCA-TCT-CAT-CCC-TGC-GTG-TCT-CCG-ACT-CAG-TAC-TGA-GCT-ACT-5GG-5CC-5YT-KCA-YAC |
| T4b | ORE | *nosZII* | nosZIIF_MID7 | CCA-TCT-CAT-CCC-TGC-GTG-TCT-CCG-ACT-CAG-CGT-GTC-TCT-ACT-5GG-5CC-5YT-KCA-YAC |
| T4c | ORE | *nosZII* | nosZIIF_MID13 | CCA-TCT-CAT-CCC-TGC-GTG-TCT-CCG-ACT-CAG-CAT-AGT-AGT-GCT-5GG-5CC-5YT-KCA-YAC |
| T5a | ORE | *nosZII* | nosZIIF_MID12 | CCA-TCT-CAT-CCC-TGC-GTG-TCT-CCG-ACT-CAG-TAC-TGA-GCT-ACT-5GG-5CC-5YT-KCA-YAC |
| T5b | ORE | *nosZII* | nosZIIF_MID21 | CCA-TCT-CAT-CCC-TGC-GTG-TCT-CCG-ACT-CAG-CGT-AGA-CTA-GCT-5GG-5CC-5YT-KCA-YAC |
| T5c | ORE | *nosZII* | nosZIIF_MID20 | CCA-TCT-CAT-CCC-TGC-GTG-TCT-CCG-ACT-CAG-ACG-ACT-ACA-GCT-5GG-5CC-5YT-KCA-YAC |

**Figure S1. Abundances of N_2_O-producers (*nirK* and *nirS*) and N_2_O –reducers (*nosZI* and *nosZII*).** Means ± sem per treatments within each experimental block are given. Significant differences between treatments are indicated with different letters (anova followed by Tukey HSD test, P<0.05).

**Figure S2. Phylogenetic placement of *nosZI* pyrosequencing reads within a reference phylogeny.** The phylogeny was inferred using maximum likelihood analysis of full-length *nosZ* amino acid sequences obtained from microbial genomes. The concentric barplots plotted around the phylogenies represent the two block experiments BE and ORE, in red and green respectively. The bars sizes correspond to the relative abundance of each OTU with respect to total read counts. Colors of branches denote taxonomic affiliation of source organisms for reference *nosZ* sequences.

**Figure S3. Phylogenetic placement of *nosZII* pyrosequencing reads within a reference phylogeny.** The phylogeny was inferred using maximum likelihood analysis of full-length *nosZ* amino acid sequences obtained from microbial genomes. The concentric barplots plotted around the phylogenies represent the two block experiments BE and ORE, in red and green respectively. The bars sizes correspond to the relative abundance of each OTU with respect to total read counts. Colors of branches denote taxonomic affiliation of source organisms for reference *nosZ* sequences.

**Figure S4. Correlation of *nosZII* phylogenetic diversity with the final denitrification product [rN_2_O/r(N_2_O+N_2_)].** Pearson correlation was calculated between Faith’s PD index of *nosZII* clade and [rN_2_O/r(N_2_O+N_2_)], (r = -0.70, P<0.0001).

## Supplementary Figures

Figure S1

**Figure S2.**

**Figure S3**

**Figure S4**


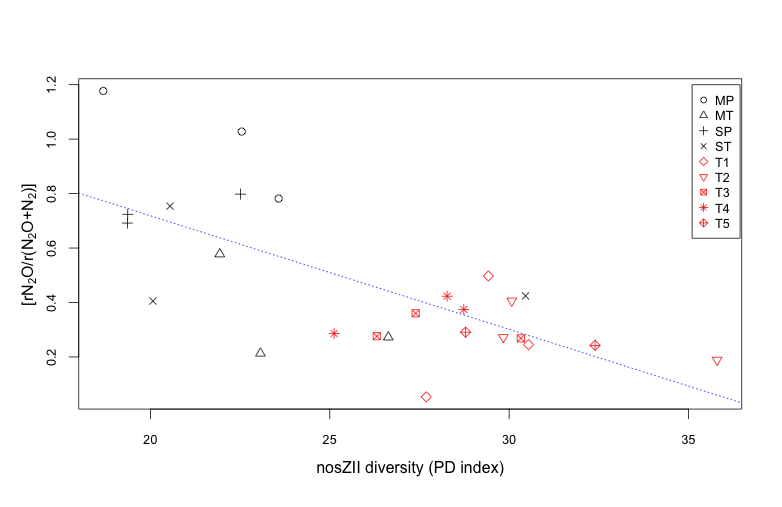

Supplement: Supplementary file 1 [file SupplementaryMaterial.DOCX]
